# Charge Transfer Complexes of Ketotifen with 2,3-Dichloro-5,6-dicyano-*p*-benzoquinone and 7,7,8,8-Tetracyanoquodimethane: Spectroscopic Characterization Studies

**DOI:** 10.3390/molecules26072039

**Published:** 2021-04-02

**Authors:** Gamal A. E. Mostafa, Ahmed Bakheit, Najla AlMasoud, Haitham AlRabiah

**Affiliations:** 1 Department of Pharmaceutical Chemistry, College of Pharmacy, King Saud University, P.O. Box 2457, Riyadh 11451, Saudi Arabia; gamostaf@ksu.edu.sa (G.A.E.M.); abakheit@ksu.edu.sa (A.B.); 2 Micro-analytical Lab., Applied Organic Chemistry Department, National Research Center, Dokki, Cairo 12622, Egypt; 3 Department of Chemistry, Faculty of Science and Technology, El-Neelain University, P.O. Box 12702, Khartoum 11121, Sudan; 4 Department of Chemistry, College of Science, Princess Nourah bint Abdulrahman University, Riyadh 11671, Saudi Arabia; nsalmasoud@pnu.edu.sa

**Keywords:** ketotifen, DDQ, TCNQ, charge transfer complex, spectroscopy, DFT

## Abstract

The reactions of ketotifen fumarate (KT) with 2,3-dichloro-5,6-dicyano-p-benzoquinone (DDQ) and 7,7,8,8-tetracyanoquinodimethane (TCNQ) as π acceptors to form charge transfer (CT) complexes were evaluated in this study. Experimental and theoretical approaches, including density function theory (DFT), were used to obtain the comprehensive, reliable, and accurate structure elucidation of the developed CT complexes. The CT complexes (KT-DDQ and KT-TCNQ) were monitored at 485 and 843 nm, respectively, and the calibration curve ranged from 10 to 100 ppm for KT-DDQ and 2.5 to 40 ppm for KT-TCNQ. The spectrophotometric methods were validated for the determination of KT, and the stability of the CT complexes was assessed by studying the corresponding spectroscopic physical parameters. The molar ratio of KT:DDQ and KT:TCNQ was estimated at 1:1 using Job’s method, which was compatible with the results obtained using the Benesi–Hildebrand equation. Using these complexes, the quantitative determination of KT in its dosage form was successful.

## 1. Introduction

Charge transfer (CT) complexes have attracted significant interest in the past few decades, owing to their wide range of applications. CT complexes are formed from the reaction of two molecules a donor and an acceptor within an intermolecular system [1,2]. The electron acceptors have high affinity, whereas the donors normally have a low ionization potential. The CT complex formation involves the excitation of an electron from the donor to an empty orbital of the acceptor [1,2]. The CT mechanism was first discovered by Mullikan [3] and later described by Foster [4]. The formation of a hydrogen bond between the donor and the acceptor plays an important role in the formation of CT complexes, and these bonds determine the stability [5,6]. 

CT complexes have many applications in different areas, such as molecular wires [7], luminescence [8], and nonlinear optics [9]. CT complexes are also applied in biological systems, such as DNA binding [10] and anti-microbial, anti-fungal, and anti-tumor mechanisms [11,12]. CT interactions are associated with the formation of colored complexes, which absorb radiation in the visible regions and can be used for the quantitative estimation of drugs [13,14]. 

Ketotifen Fumarate (KT) also known as 10 H-benzo [4,5] cyclohepta [1, 2-b] thiophen-10-one,4,9-dihydro-4-(1-methyl-4-piperidinylidene), (2E)-2-butenedioate is a is a non-specific mast cell stabilizer [15]. The primary biochemical and pharmacological activity of KT is exerted by acting as a H1-receptor antagonist and phosphodiesterase inhibitor, leading to the inhibition of calcium flux in smooth muscle. Different analytical techniques have been reported to determine KT [16,17,18,19,20,21], but most are time-consuming, costly, or require derivatization. CT complexes in combination with spectrophotometry represent a cheaper and faster alternative for the quantification of KT based on a colorimetric measurement.

This study aimed to investigate the stability of the formed of charge transfer complexes based on a spectrophotometric study of KT with the acceptors (DDQ and TCNQ). A number of different spectroscopic factors were estimated, including the formation constant, molar absorptivity, oscillator strength, dipole moment, ionization potential, resonance energy, dissociation energy, and standard free energy of complexation. It aimed to achieve the spectrophotometric determination of KT based on the CT complexes. Density function theory (DFT) was used to characterize the molecules in the complexes based on their electronic structures and physicochemical properties, such as charge distribution, molecular electrostatic potential, energy variance among the highest occupied molecular orbital (HOMO) and lowest unoccupied molecular orbital (LUMO), and the electronic chemical potential analysis of the CT complexes. DFT has previously been carried out to describe the physiochemical properties of various CT complexes [13,14] and is one of the most useful quantum calculation methods available, due to its reliability, low cost, and speed.

## 2. Results and Discussion

**Figure 1:**
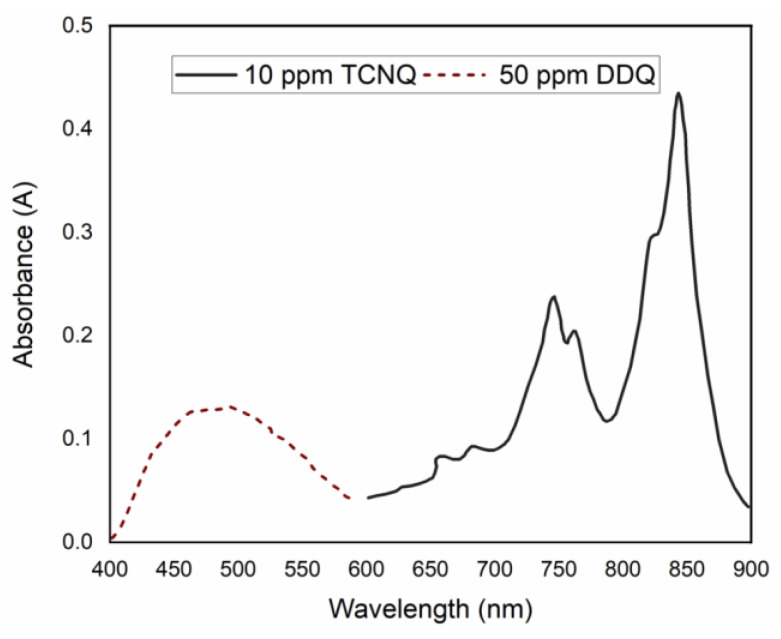
Absorbance curves of KT-DDQ and KT-TCNQ complexes.

In this work, the reaction of KT with DDQ or TCNQ to produce CT complexes was investigated. Previously, the concepts underlying the formation of CT complexes between electron donors and acceptors have been explored in detail [13,14]. The major chromogen with DDQ has a maximum absorbance in acetonitrile of 485 nm in the visible region. Meanwhile, the major product of KT with TCNQ is a green radical anion, which is characterized by a maximum absorabance of 843 nm (Figure 1).

### 2.1. Molar Composition of KT Complexes

**Figure 2:**
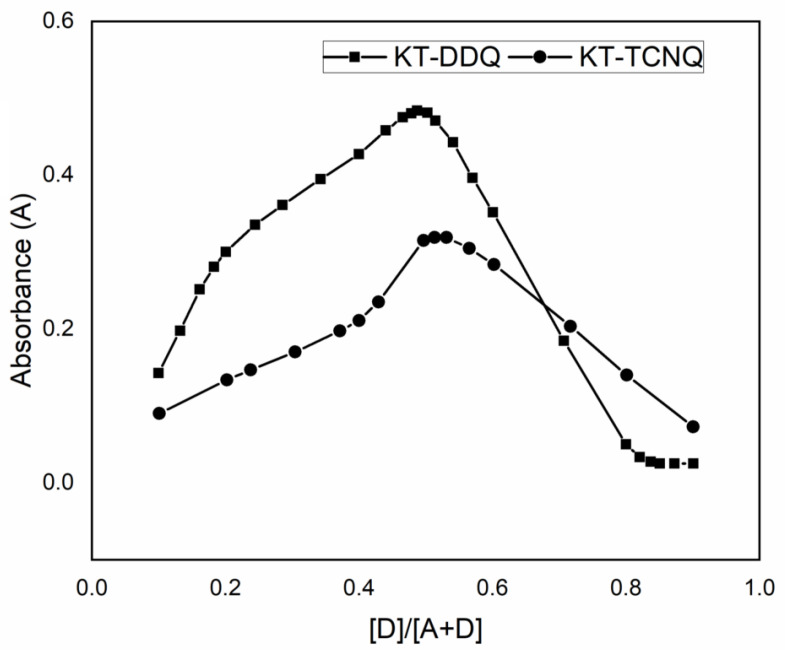
Job’s method to determine K:DDQ and KT:TCNQ ratios.

**Figure 3:**
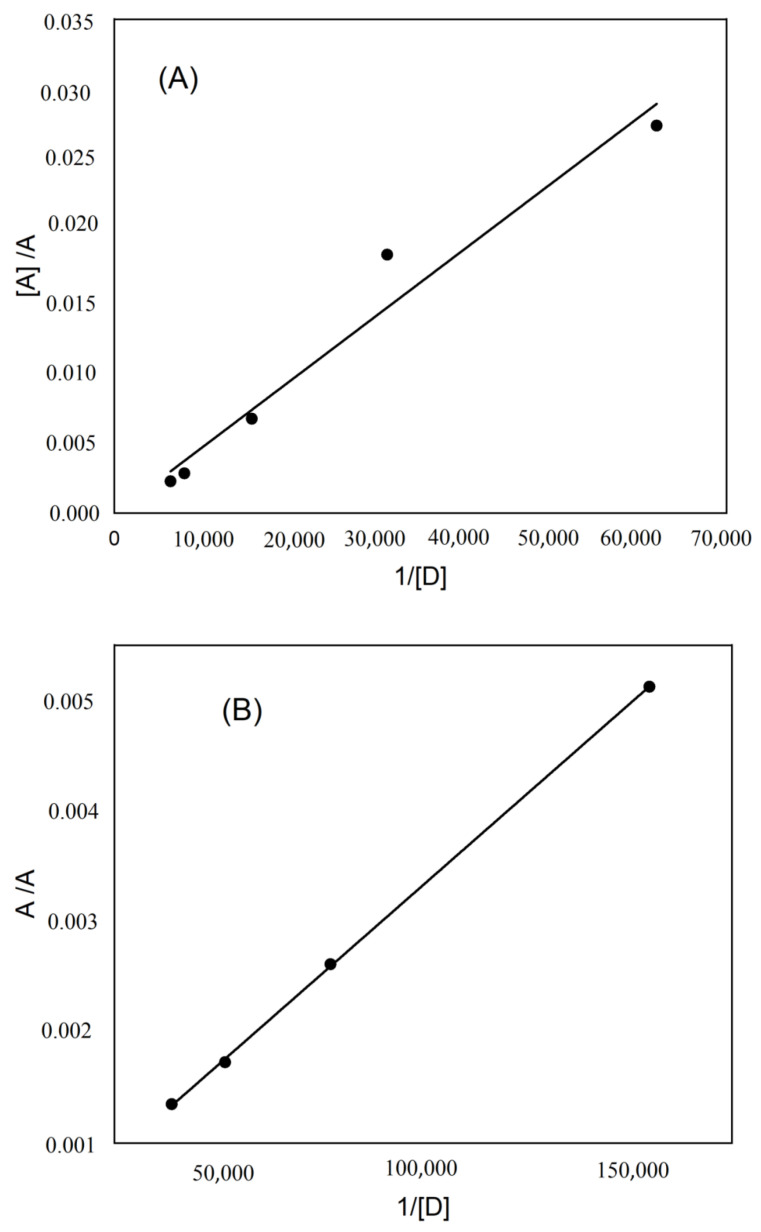
The Benesi–Hildebrand plot for the (**A**) KT-DDQ and (**B**) KT-TCNQ complexes.

The stoichiometry of the produced complexes was evaluated using Job’s method [22]. It was found that KT reacted with DDQ or TCNQ in a 1:1 ratio (Figure 2), which agrees with the results obtained by the modified Benesi–Hildebrand equation. The modified Benesi–Hildebrand equation [23] was used to resolve the formation constants of the complexes according to the following equation: $\frac{\left[A\right]}{A}=\frac{1}{\varepsilon_{AD}}+\;\frac{1}{K_{C}\;\varepsilon }\;l/\left[D\right]\;$ [23], where the acceptor (DDQ or TCNQ) molar concentration is denoted by [*A*], the donor’s molar concentration (KT) is denoted by [D], the absorbance of the formed complex at λ_max_ is denoted by *A*, the complex’s molar absorptivity is denoted by ε, and the association constant of the complex is denoted by K_C_. After plotting the values of [*A*]/*A* against l/[D], a straight line was fitted (Figure 3). From the trend line function, the coefficients for the determination (*r*^2^), association constant (K_c_), and molar absorptivity (ε) of the KT→DDQ and KT→TCNQ complexes were calculated, where ε was determined from the inverse of the intercept value and the association constant (*K_C_*) was determined from the slope (1/ε *K_C_* is the slope). Table 1 shows the Benesi–Hildebrand data. The ε_*AD*_ and *K_C_* of the formed complexes were found to be (1.1 × 10^3^ and 1.3 × 10^4^ L mol^−1^ cm^−1^) for KT-DDQ and (0.2 × 10^3^ and 2.56 × 10^3^ L mol^−1^ cm^−1^) for KT-TCNQ. The high value of the association constant confirms the stability of the CT complexes, which is in line with the published literature on the association constants [13,14]. The stability of the formed 1:1 complex was higher with KT-TCNQ than with KT-DDQ.

### 2.2. The Determination of Spectroscopic Physical Parameters

The oscillator strengths (f) [10,12] and transition dipole moments (μ, Debye) [12] of each complex were determined according to the equations $f=4.32\times 10^{-9}\left[\varepsilon_{max}\;\Delta v_{1/2}\right]$ [10,12] and $=0.0958\;{\left[\frac{\varepsilon_{max\Delta v_{\frac{1}{2}}}}{{\bar{v}}_{max}}\right]}^{1/2}$ [12], where $\Delta v_{1/2}$ is the bandwidth at half intensity and ${}_{max}$ and ${\bar{v}}_{max}$ correspond to the molar extinction coefficient and wavenumber at the highest absorbance of the compound, respectively. The values are presented in Table 2. The relatively high values of f (0.34 for KT-DDQ and 9.36 for KT-TCNQ) indicate a strong interaction between KT and DDQ or TCNQ, and therefore the high stability of the complexes (KT-DDQ and KT-TCNQ). Furthermore, the transition dipole moments showed high values, 5.9 (KT-DDQ) and 12.9 (KT-TCNQ), indicating the creation of strong bonds between the donor and acceptors. 

The following equation was applied to calculate the donor’s ionization potentials (Ip) in the CT [24]: $Ip\;\left(eV\right)=5.76\;+\;1.53\;\times \;10^{-4}\;v_{CT}\;$ [24], where $v_{CT}$ corresponds to the wavenumber in cm^−1^, equivalent to the CT band of the formed complexes. The Ip values are listed in Table 2. Ip is measured using the electron-donating power of a donor molecule—i.e., the energy of ionization that is needed to remove an electron from its molecule. The energies of the CT complexes (*E_CT_*) were analyzed, as reported in [10], according to the equation below, and their values are shown in Table 2. $E_{CT}=\;hv_{CT}=1243.667{/}_{CT}$, where λ_CT_ is related to the wavelength of the complexation bands. The resonance energies (R_N_) of the CT complexes were assessed using the Briegleb and Czekalla equation [25], and their values are presented in Table 2. ${}_{CT}=\;7.7\times 10^{-4\;}/\left[hv_{CT}/R_{N}\right]-3.5$ [25], where ε_CT_ corresponds to the molar absorptivity of the CT complexes. The stability of the CT complexes in the ground state is indicated by the values of the resonance energy. The dissociation energy (W) was used to express the nature of the CT complex in the excited state. The dissociation energy was calculated using the following equation and the values are presented in Table 2. $W=I_{P}-\;E^{A}-E_{CT\;}$, where *E_CT_* is the energy of the CT complex, *Ip* is the ionization potential of the donor, and E^A^ is the electron affinity of the acceptors.

The nature of the CT interactions was indicated by the value standard free-energy change (Δ*G*°), presented in Table 2. Negative values indicate that the reactions between KT and the acceptors are exothermic. As the values become more negative, the formation constant increases [10]. The standard free-energy change was computed according to the equation below: $\Delta G^{\circ }=-\mathrm{RT}\;\ln\;K_{CT}$ [10], where Δ*G* is the free-energy change of the CT complex (kJ mol^−1^), R is the gas constant (1.987 cal mol^−1^ K^−1^), T indicates the absolute temperature in Kelvins, and K_CT_ is the association constant of the CT complex at room temperature. The negative values of free energy indicate spontaneous reactions between the donor and acceptor. The Δ*G*° values were in the following order: KT-DDQ > KT-TCNQ.

### 2.3. Validation

#### 2.3.1. The Lower Limit of Detection and Quantification

Two calibration graphs were obtained under optimal reaction conditions by plotting the average absorbance against the KT concentration. Two linear equations were obtained by regression analysis, expressed as (y = *a*x ± *b*), where y is the absorbance, x is the concentration, *a* is the slope, and *b* is the intercept. The regression correlation coefficient *r*^2^ was assessed. Table 3 shows the analytical parameters of the developed method. Beer’s law linearity was demonstrated over ranges of 5–100 and 2.5–40 ppm for DDQ and TCNQ, respectively.

The lower limit of detection (LOD) and quantification (LOQ) were calculated [26]. The LOD was set to equal 3.3 times the level of the noise, while the LOQ was set to equal 10 times the noise based on six replicate measurements. The LOQ was 5.0 and 2.5 ppm and the LOD was 1.5 and 0.75 ppm for KT-DDQ and KT-TCNQ, respectively.

#### 2.3.2. Precision and Accuracy

The accuracy, intra-, and inter-day precision of the KT assay using either KT-DDQ or KT-TCNQ were investigated by the developed method (*n* = 6).

The expressions of precision and accuracy are denoted as RSD and % recovery, respectively. The obtained results (Table 4) were in the acceptable range; the accuracy was approximately 98.0%, while the precision (relative error) was less than 3.0%.

### 2.4. DFT/TD-DFT Calculations

#### 2.4.1. Optimized Geometrical Structures

**Figure 4:**
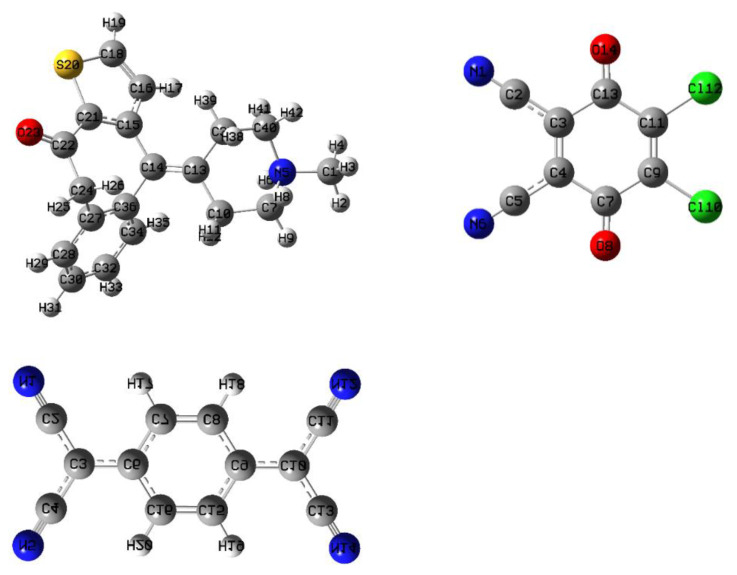
Optimized structures of KT, DDQ, and TCNQ.

The full geometry optimization of the donor (KT), π-acceptors (DDQ and TCNQ), and CT complexes (KT-DDQ and KT-TCNQ) was carried out using the ChembioDraw Ultra software. Subsequently, the bond lengths between the constituent atoms were analyzed employing the computational methods of DFT/MPW1PW91 with a 6–311 G(d,p) basis set. The molecular structures of the complexes, atom numbering, and the hydrogen bonding interactions are presented in Figure 4.

The bond lengths increased in the C18-N19, C41-C42, C44-Cl52, and C46-O47 bonds in KT, DDQ, and KT-DDQ from 1.4543, 1.347, 1.7008, and 1.2031 Å in the optimized structure of free compounds to 1.4703, 1.4175, 1.7353, and 1.2523 in the optimized structure of the complex, respectively, as shown in Table S1. This is attributed to a resonance effect and the formation of the hydrogen bonds N19....O47 with a bond length of 3.253 Å. On the other hand, the bond lengths of N19-C21 and C42-C43 in KT, DDQ, and KT-DDQ were 1.4592 and 1.4959 Å, respectively, in the optimized structure of free compounds, while this decreased to 1.2807 and 1.3855 Å, respectively, in the optimized structure of the complex, as shown in Table S1. This is due to a resonance effect. In the interaction between KT with TCNQ, the bond lengths of the C5-C11 and C6-C12 bonds of TCNQ increased from 1.3792 to 1.4214 and 1.423 Å, respectively, in the optimized structure of the KT-TCNQ complex, as shown in Table S1. This again is attributed to a resonance effect. Finally, the bond length of the N25-C26 and C26-C28 bonds in the optimized structure of TCNQ decreased from 1.4543 and 1.5327 Å to 1.3841 and 1.4879 Å, respectively, in in the optimized structure of the KT-TCNQ complex, as shown in Table S1. This is also due to a resonance effect.

**Figure 5:**
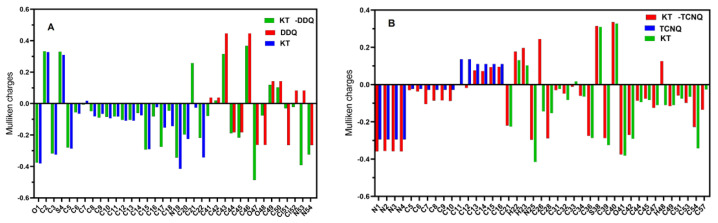
(**A**) The Mulliken charge and natural atomic charge distribution of the (KT–DDQ) complex and (**B**) the Mulliken charge and natural atomic charge distribution of the (KT–TCNQ) complex by the DFT/MPW1PW91 methods with a 6-311G(d, p) basis set.

Mulliken atomic charges of KT, DDQ, and TCNQ and their complexes were calculated by the MPW1PW91/6-311G (d, p) method [27]. Figure 5 and Table S2 show the Mulliken atomic charges plot and their values, respectively.

**Scheme 1:**
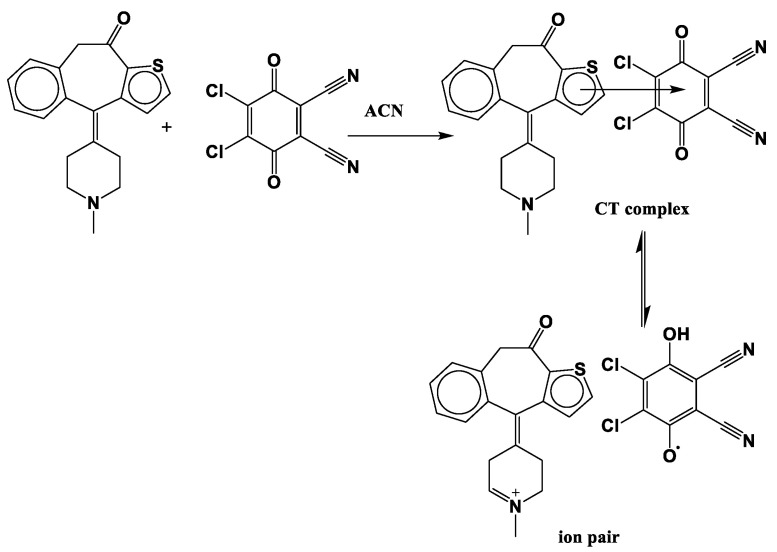
Reaction mechanism of the KT-DDQ complex.

**Scheme 2:**
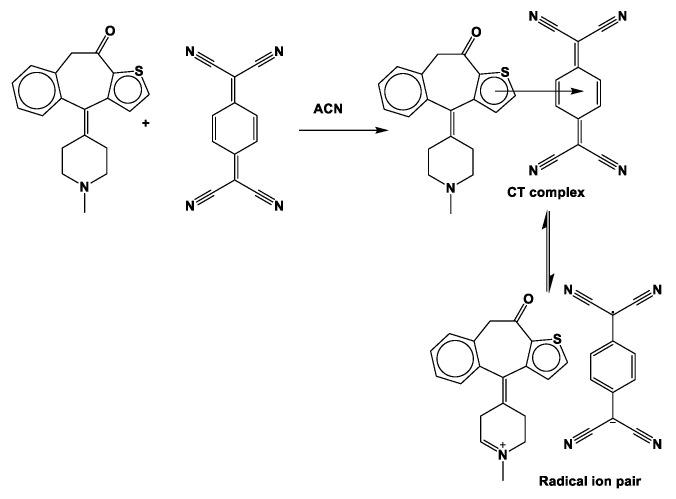
Reaction mechanism of the KT-TCNQ complex.

In the case of the KT-TCNQ complex, all carbon atoms are negatively charged except C13 to C16, C26, and C40, owing to an electron attracting electronegative oxygen or nitrogen atoms, as shown in Table S2. The oxygen atoms in KT-DDQ have negative Mulliken charges and act as an electron acceptor. In KT and KT-TCNQ, the C33 and C40 atoms have a positive charge, while the C33 atom has a negative charge in the KT-TCNQ complex. In addition, the atom with the highest negative charge is the C28 atom. In KT and KT-DDQ, the C2, C41, C42, C43, C44, C46, C49, and C50 atoms have negative charges. Moreover, in all compounds, most of the positive charges are on hydrogen atoms. When combining the acceptor and donor at low concentrations, a reddish-brown color was visibly detected, indicating the formation of the CT complex. The donating nature of KT due to the presence of a nitrogen center, as well as the high electron affinity of DDQ and TCNQ, are responsible for generating the radical anion. This is the hallmark of the formation of a radical ion pair resulting from an electron transfer from KT towards DDQ [28] (Scheme 1 and Scheme 2).

**Figure 6:**
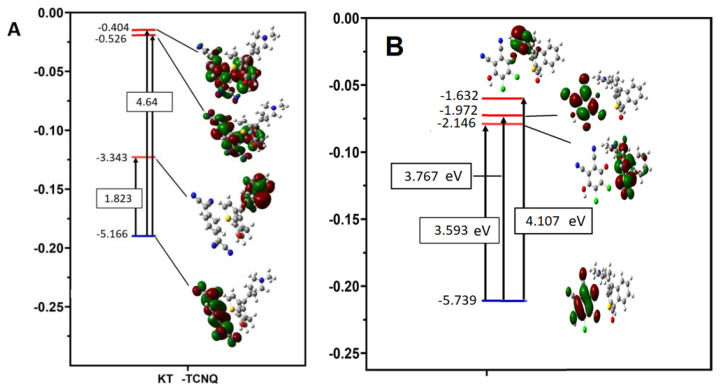
The assignments of theoretical electronic absorption transitions of (**A**) KT-DDQ and (**B**) KT-DDQ charge transfer complexes.

The characteristics of the electronic absorption transition monitored in the CT complexes were assigned by the computational methods of TD-DFT/MPW1PW91 with a 6-311G(d,p) basis set in the presence of acetonitrile [29,30,31]. Figure 6A,B show various HOMOs and LUMOs of the KT-DDQ and KT-TCNQ complexes in the ground state. The energies of HOMO, HOMO^−n^, LUMO, and LUMO^+n^, where *n* = 1–4, for the KT, DDQ, TCNQ, KT-TCNQ, and KT-DDQ complexes in both the ground and excited states are provided. The HOMO and LUMO energies for the KT-DDQ and KT-TCNQ complexes, based on the optimized structure, were computed at −5.739 to −2.146 eV and −5.166 to −3.343 eV, respectively. HOMO and LUMO of the KT-DDQ and KT-TCNQ complexes in the ground state were obtained using the same level of theory optimization. Figure 6 indicates that HOMO is localized on KT only while LUMO is localized on the DDQ or TCNQ moieties. As presented in Figure 6, the red and green colors denote the positive and negative stages, respectively, and among all compounds, the KT-TCNQ and KT-DDQ complexes have smaller energy gaps, at 1.824 and 3.592 eV, than those of their reactants, meaning that these complexes show more stability and strength than their reactants. The ionization potential (Ip) values for KT, DDQ, TCNQ, KT-TCNQ, and KT-DDQ indicate that energies of 6.38, 6.58, and 7.09 eV are required to retrieve an electron from the HOMO orbitals, suggesting that it is harder to remove an electron from the DDQ and TCNQ. In addition, among these compounds, KT has the lowest electron affinity (1.975 eV) and the highest molecular reactivity to nucleophiles. The chemical hardness (η) values of these compounds decrease in the order of KT, DDQ, TCNQ, KT-DDQ, and KT-TCNQ, while the softness values increase in the same order (Table 5). In other words, lower softness and higher chemical hardness confirm the higher stability of the KT-TCNQ and KT-DDQ complexes compared to their reactants (donor and acceptor), which are thought to have high molecular hardness. The dipole moments of the KT-TCNQ and KT-DDQ complexes are calculated as 0.912 and 1.796 Debye, respectively (Table 5). The outcome shows that the complexes have stronger intermolecular interactions than the other compounds and interact with each other more than with the surrounding environment.

**Figure 7:**
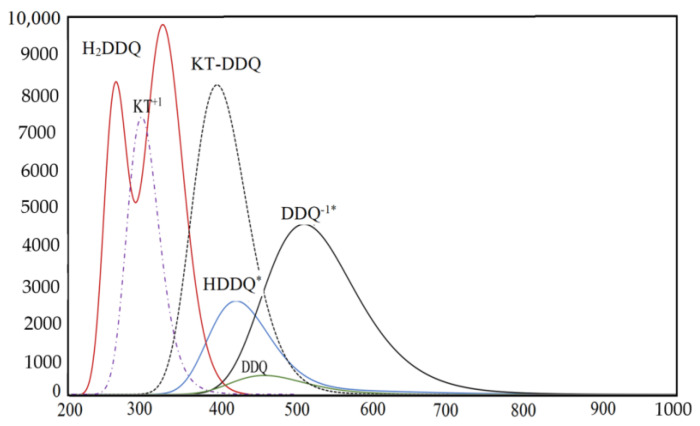
The electronic absorption spectra of the donor (KT) and acceptor DDQ as well as their probability ions (DDQ^−1^·, HDDQ·, and H2DDQ) and CT complex (KT–DDQ) in ACN at 298 K.

**Figure 8:**
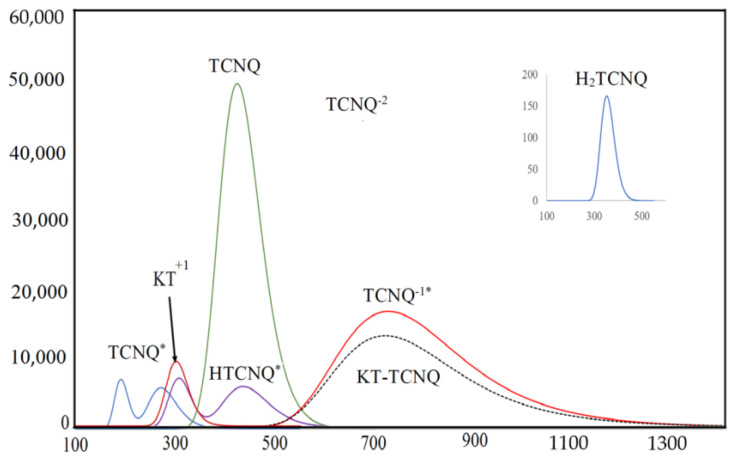
The electronic absorption spectra of the donor (KT) and acceptor (TCNQ), as well as their probability ions (TCNQ·, HTCNQ·, TCNQ^−2^, TCNQ^−1^·, and H_2_TCNQ) and CT complex (KT–TCNQ) in ACN at 298 K.

The absorption spectra of the donor (KT), acceptor (DDQ and TCNQ), and CT complexes are displayed in Figure 7 and Figure 8. Multicharge transfer bands at 588, 546, and 457 nm in the spectra of the complexes do not exist in the spectra of free donor or free acceptor absorption spectra are displayed in Figure 7 and Figure 8 and Table S3. Multicharge transfer bands at 424.71, 378.37, and 285.17 nm do not exist in the spectra of the free donors or free acceptors. The transitions relate to the electronic transitions from the HOMO to the LUMO [32].

#### 2.4.2. Molecular Electrostatic Potential (MEP) Surface Analysis

**Figure 9:**
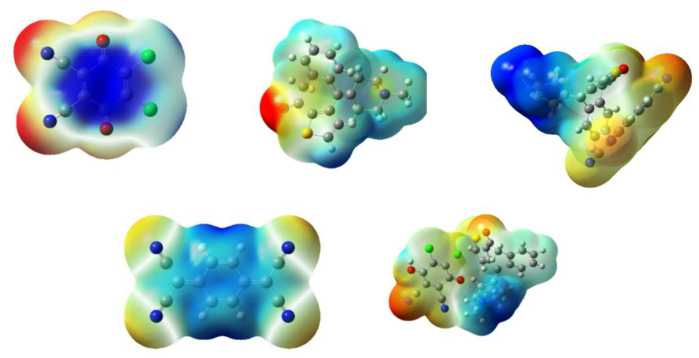
Molecular electrostatic potential maps of KT, DDQ, TCNQ, KT→DDQ and KT→TCNQ (the red color represents maximum negative area or a favorable site for an electrophilic attack, the blue color indicates the maximum positive area or a favorable site for a nucleophilic attack, and the green color represents zero potential area).

The molecular electrostatic potential map of a compound [33] is useful in investigating the reactive sites of the molecules—i.e., nucleophilic or electrophilic attack zones as well as hydrogen bonds [34,35]. Moreover, the electrostatic potential (ESP) is linked to the partial charges, dipole moments, electronegativity, and chemical reactivity zones of the molecules. Figure 9 presents the MEP map for KT, DDQ, TCNQ, and their complexes. The electrostatic potential from the total self-consistent field (SCF) density, which does not have a gradient of electrostatic potential, shows that negative ESP in KT was situated over the oxygen atom of the carbonyl group. On the other hand, in DDQ, this was positioned over the nitrogen atom and slightly over the oxygen atom of the carbonyl group. In TCNQ, this was primarily over the nitrogen atom. The negative ESPs appears as yellow spots on a reddish surface or as reddish spots on a yellow surface. In addition, the contour surfaces (yellow and red lines) confirm positive and negative potential regions, and they are in agreement with the ESP data. These regions indicate favored sites for electrophilic attacks towards the molecule.

#### 2.4.3. Thermodynamics of the KT-DDQ and KT-TCNQ Complexes

Thermodynamic parameters, such as enthalpy change (H^0^), entropy change (S^0^) and free energy change (G^0^), were computed at 298.15 K and 1.00 atm pressure by HF/MPW1PW91/6−311G (d, p) [29,30], and their values are shown in Table 6. Regarding the outcomes achieved from the calculation of statistical thermodynamics, it was observed that the charge transfer reaction is exothermic in the KT-DDQ complex and endothermic in KT-TCNQ, which is consistent with the outcomes of the experiments.

### 2.5. Applications

The proposed spectrophotometric techniques were carried out for the determination of KT in synthetic dosage form. The data showed an average recovery of 97.5% with a relative standard deviation (error) of less than 3%. The performance results matched those obtained by a previously reported HPLC method [18], which achieved a high recovery of 106% with an RSD of 2.37%.

## 3. Experimental

### 3.1. Apparatus

A double beam ultraviolet-visible spectrophotometer (Thermo Scientific, Loughborough, UK) was carried out with a wavelength range of 200 to 900 nm, a spectral bandwidth of 2 nm, and 1 cm quartz cells.

### 3.2. Reagents and Materials

Ketotifen fumarate (KT), a pure analytical reagent, was obtained from Sigma (Saint Louis, MS, USA). Deionized water was used in all experiments. 2,3-Dichloro-5,6-dicyano-p-benzoquinone (DDQ) and 7,7,8,8-tetracyanoquinodimethane (TCNQ) were purchased from Sigma-Aldrich (Steinheim, Germany). The stock solution of KT (10 mg/mL) was prepared in acetonitrile and the working solution (1000 μg/mL) was obtained through the further dilution of the stock solutions by acetonitrile.

### 3.3. Procedure

One milliliter of the KT solution (containing 100–1000 μg/mL) or (25–400 μg/mL) was transferred into 10 mL flasks, then 1 mL of the acceptors (DDQ and TCNQ) (1 mg/mL) was added, and the volume was made up with acetonitrile. The resultant red color was measured by spectrophotometry at 485 nm. In the case of TCNQ, the color was measured at 843 nm after heating at 70 °C for 20 min, against a reagent blank prepared by the same way without KT. Using serial dilutions, calibration curves were constructed (absorbance vs. concentration of KT) for both complexes.

### 3.4. Determination of the Molar Ratio in TK-DDQ and TK-TCNQ Complexes

Job’s system of continuous variation [22] was applied using a master concentration of KT and DDQ or TCNQ (6.46 × 10^−4^ M). A set of experiments were conducted by mixing different complementary ratios of the master solutions (1:9, 2:8, up to 8:2, 9:1, inclusive) in a 10 mL measuring flask. The solutions were treated and measured as detailed in the general procedure (Section 3.3).

### 3.5. Conductance Measurements

The conductivities of ketotifen and its CT complex (KT-DDQ and KT-TCNQ) was measured in acetonitrile (0.6 mg/mL) using an Orion conductometer (Beverly, MA 01915-6199, USA). The conductance was 19, 88, and 67 µS for KT, KT-DDQ, and KT-TCNQ, respectively. These values indicate the electrolytic nature of the formed complexes.

### 3.6. Computational Details

Computational density functional theory (DFT) was used with the MPW1PW91 function and the “6–311++G(d,p)” basis set for KT, DDQ, TCNQ, and the KT–DDQ and KT–TCNQ complexes to investigate the perfect ordinary style investigation and geometry optimization of the complexes [36,37]. Time-dependent “DFT” calculations were performed by “MPW1PW91/6-31G (d,p)” methods in acetonitrile using the default polarizable continuum model. The DFT calculations were carried out by the Gaussian 09W package (340 Quinnipiac St Bldg 40, Wallingford, CT 06492, USA) [36,38].

## 4. Conclusions

The CT complexes of KT with DDQ and TCNQ were investigated using spectrophotometric and theoretical studies. The proposed reactions resulted in the successful determination of KT in its dosage from. The molar ratio was assessed using Job’s method, which was in agreement with the findings of the Benesi–Hildebrand equation. A number of key spectroscopic parameters of CT complexes were assessed. In addition, DFT allowed for geometry optimization, charge distribution elucidation, the investigation of molecular electrostatic potential, HOMO–LUMO calculations, and the estimation of thermodynamics parameters. As a result of these findings, a complete and reliable assessment was achieved for the studied CT complexes.

## Figures and Tables

**Table 1:** Benesi–Hildebrand data for the KT–DDQ and KT–TCNQ complexes.

[*A*], M	[D], M	Absorbance (A)	1/[D]	[*A*]/*A*
KT–DDQ				
0.00044	1.62 × 10^−5^	0.016	61,885.2	0.0275
0.00044	3.23 × 10^−5^	0.024	30,942.6	0.018333
0.00044	6.46 × 10^−5^	0.065	15,471.3	0.006769
0.00044	0.00012927	0.157	7735.65	0.002803
0.00044	0.00016159	0.196	6188.52	0.002245
KT–TCNQ				
0.000489	6.46 × 10^−5^	0.095	154,712.5	0.005147
0.000489	1.293 × 10^−5^	0.186	77,356.25	0.002629
0.000489	1.94 × 10^−5^	0.28	51,570.83	0.001746
0.000489	2.585 × 10^−5^	0.36	38,678.13	0.001358

**Table 2:** Spectroscopic parameters for KT-DDQ and KT-TCNQ.

Parameters	KT-DDQ	KT-TCNQ
Wavelength, nm	485	843
Extinction coefficient: ε = L mol^−1^ Cm^−1^	1.1 × 10^3^	1.3 × 10^4^
Association constant: K = L mol^−1^	0.2 × 10^3^	2.56 × 10^3^
Energy: *hv* (eV)	2.56	1.47
Ionization Potential: Ip (eV)	8.91	7.57
Resonance energy: R_N_ (eV)	3.5	3.5
Dissociation energy: W (eV)	3.28	3.32
Gibbs free energy: Δ*G* (kJ mol^−1^)	−4.5 × 10^3^	−4.6 × 10^3^
*F*	0.34	5.91
*µ*	5.91	12.9

**Table 3:** Spectrophotometric characterization parameters for the KT-DDQ and KT-TCNQ complexes.

Parameters	KT-DDQ	KT-TCNQ
Wavelength, nm	485	843
Calibration range	5–100	2.5–40
Extinction coefficient: ε (L mol^−1^ cm^−1^)	1.1 × 10^3^	1.3 × 10^4^
Lower limit of quantification	5	2.5
Lower limit of detection	1.5	0.75
Slope	0.0042	0.0321
Intercept	0.0126	0.0741
Correlation coefficient, *r*^2^	0.998	0.998

**Table 4:** Accuracy and precision of the proposed method.

Conc, μg/mL		Intra-Day Recovery % ± RSD		Inter-Day Recovery % ± RSD	
KT-DDQ	KT-TCNQ	KT-DDQ	KT-TCNQ	KT-DDQ	KT-TCNQ
15	10	97.5 ± 2.7	97.0 ± 2.7	97.0 ± 2.9	97.5 ± 2.9
35	20	97.5 ± 2.6	97.5 ± 2.5	97.5 ± 2.9	98.0 ± 2.8
75	30	98.0 ± 2.5	98.0 ± 2.5	98.0 ± 2.8	98.0 ± 3.0

**Table 5:** The electronic reactivity descriptors of the KT, DDQ, TCNQ, KT-TCNQ, and KT-DDQ.

Parameters (eV)	DDQ	TCNQ	KTN	KTN-TCNQ	KTN-DDQ
E_LUMO (eV)_	−4.804	−4.529	−1.975	−3.343	−2.147
E_HOMO (eV)_	−8.409	−7.419	−6.205	−5.167	−5.739
Energy band gap(eV) [∆E = E_LUMO_ − E_HOMO_]	3.605	2.89	4.23	1.824	3.592
Chemical hardness (η)	1.802	1.445	2.115	0.912	1.796
Softness (ς)	0.555	0.692	0.473	1.097	0.557
Dipole moment (µ) (Debye)	1.802	1.445	2.115	0.912	1.796
Electrophilicity index (ω)	0.901	0.723	1.058	0.456	0.898
Ip(eV)	8.409	7.419	6.205	5.167	5.739
Electron affinity (EA)	4.804	4.529	1.975	3.343	2.147
Electronegativity (χ)	6.607	5.974	4.09	4.255	3.943

**Table 6:** The statistical thermodynamic calculation for the KT-DDQ and KT-TCNQ complexes.

CT Complex	G^0^ (kcal/mol)	H^0^ (kcal/mol)	S^0^ (cal/K mol)
KT-DDQ	−18.1827	−30.1029	−0.04
KT-TCNQ	389.4368	359.9338	−0.099

## Data Availability

Not applicable.

## Supplementary Materials

The following are available online. Figure S1: Bond lengths (Å) for DDQ, TCNQ, KT, KT→TCNQ and KT→DDQ complexes estimated using DFT (6-311G (d,p) basis set) calculation, Table S1: Bond lengths (Å) for DDQ, TCNQ, KT, KT→TCNQ and KT→DDQ complexes estimated using DFT (6-311G (d,p) basis set) calculation, Table S2: Mulliken atomic charges for DDQ, TCNQ, KT, KT→TCNQ and KT→DDQ complexes, Table S3: Calculation UV-Vis of KT, KT^+1^, DDQ, HDDQ·, TCNQ, TCNQ^−1^·, KT^+1^→TCNQ^−1^· and KT^+1^→HDDQ· by TD—DFT/ MPW1PW91 methods with 6-311++ G(d,2p) basis sets.

## Abbreviations

CT	Charge transfer
KT	Ketotifen Fumarate
DDQ	2,3-dichloro-5,6-dicyano-p-benzoquinone
TCNQ	7,7,8, 8-tetracyanoquinodimethane
DFT	density functional theory
f	oscillator strength
μ	transition dipole moment
Ip	ionization potentials
$E_{CT}$	energy of charge transfer complex
W	dissociation energy
Δ*G*	Gibbs free energy
R_N_	Resonance energy
ε	extinction coefficient
MEP	molecular electrostatic potential
HOMO	highest occupied molecular orbital
LUMO	lowest unoccupied molecular orbital
