# Supplementary material for: Charge Transfer Complexes of Ketotifen with 2,3-Dichloro-5,6-dicyano-p-benzoquinone and 7,7,8,8-Tetracyanoquodimethane: Spectroscopic Characterization Studies
Source: Molecules. 2021 Apr 2;26(7):2039. doi: 10.3390/molecules26072039 (PMC8038309; doi:10.3390/molecules26072039)
Supplement: Supplementary file 1 [file molecules-26-02039-s001.pdf]

Table S1: Bond lengths (Å) for DDQ, TCNQ, KT, KT→TCNQ and KT→DDQ complexes estimated using DFT (6-311G (d,p) basis set) calculation.

	DDQ			TCNQ			
Bond length	KT→DDQ	KT	DDQ	Bond length	KT→TCNQ	TCNQ	KT
O1-C2	1.2188	1.223		N1-C13	1.158	1.154	
C2-C3	1.4596	1.4562		N2-C14	1.158	1.154	
C2-C15	1.5166	1.5164		N3-C15	1.158	1.154	
C2-O47	4.9508			N4-C16	1.1581	1.154	
C3-S4	1.7298	1.7353		C5-C7	1.4301	1.4413	
C3-C7	1.3862	1.3881		C5-C8	1.4301	1.4414	
S4-C5	1.7079	1.7098		C5-C11	1.4214	1.3792	
C5-C6	1.3658	1.3655		C6-C9	1.4294	1.4413	
C5-H23	1.0794	1.08		C6-C10	1.4299	1.4413	
C6-C7	1.4212	1.4232		C6-C12	1.423	1.3792	
C6-H24	1.0807	1.0812		C7-C9	1.3879	1.3472	
C7-C8	1.4784	1.4778		C7-H17	1.0936	1.0823	
C8-C9	1.4898	1.4919		C8-C10	1.3878	1.3472	
C8-C16	1.3444	1.3474		C8-H18	1.0931	1.0823	
C9-C10	1.4009	1.4008		C9-H19	1.0945	1.0823	
C9-C14	1.394	1.3948		C10-H20	1.0932	1.0823	
C10-C11	1.3921	1.3933		C11-C13	1.4265	1.4202	
C10-C15	1.5062	1.5065		C11-C14	1.4265	1.4202	
C11-C12	1.3903	1.3903		C12-C15	1.4264	1.4202	
C11-H25	1.0842	1.0846		C12-C16	1.4262	1.4202	
C12-C13	1.3886	1.39		H19-C45	2.8519		
C12-H26	1.0833	1.0837		C21-H22	1.1026		1.0919
C13-C14	1.3901	1.3904		C21-H23	1.1017		1.1042
C13-H27	1.0833	1.0837		C21-H24	1.1027		1.0927
C14-H28	1.0838	1.0842		C21-N25	1.488		1.4473
C15-H29	1.0967	1.0968		N25-C26	1.3841		1.4543
C15-H30	1.0888	1.0891		N25-C57	1.4922		1.4592
C16-C17	1.4985	1.5088		C26-H27	1.0772		1.0915
C16-C22	1.5011	1.4988		C26-C28	1.4879		1.5327
C17-C18	1.5201	1.5327		C28-H29	1.1133		1.0971
C17-H31	1.0958	1.0971		C28-H30	1.1179		1.089
C17-H32	1.0878	1.089		C28-C31	1.5082		1.5088
C18-N19	1.4703	1.4543		C31-C32	1.3523		1.3474

C18-H33	1.0896	1.1048		C31-C54	1.5017		1.4988
C18-H34	1.0924	1.0915		C32-C33	1.4673		1.4778
N19-C20	1.4602	1.4473		C32-C53	1.4828		1.4919
N19-C21	1.2807	1.4592		C33-C34	1.4486		1.4232
N19-O47	3.253			C33-C39	1.3786		1.3881
C20-H35	1.0899	1.0919		C34-H35	1.0849		1.0812
C20-H36	1.0866	1.1042		C34-C36	1.3612		1.3655
C20-H37	1.0894	1.0927		C36-H37	1.082		1.08
C21-C22	1.5367	1.5367		C36-S38	1.738		1.7098
C21-H38	1.0943	1.0943		S38-C39	1.7359		1.7353
C22-H39	1.0993	1.0967		C39-C40	1.4546		1.4562
C22-H40	1.0925	1.0882		C40-O41	1.222		1.223
H29-O47	4.0062			C40-C42	1.5151		1.5164
C41-C42	1.4175		1.347	C42-H43	1.1128		1.0891
C41-C46	1.4322		1.496	C42-C44	1.5008		1.5065
C41-C49	1.4097		1.4207	C42-H60	1.1178		1.0968
C42-C43	1.3855		1.4959	C44-C45	1.4009		1.3933
C42-C50	1.4199		1.4209	C44-C53	1.4121		1.4008
C43-C44	1.4095		1.4856	C45-H46	1.0908		1.0846
C43-O48	1.3473		1.2031	C45-C47	1.3981		1.3903
C44-C45	1.3788		1.3475	C47-H48	1.0904		1.0837
C44-Cl52	1.7353		1.7008	C47-C49	1.3974		1.39
C45-C46	1.4497		1.4855	C49-H50	1.0903		1.0837
C45-Cl51	1.7234		1.7011	C49-C51	1.3977		1.3904
C46-O47	1.2523		1.2031	C51-H52	1.0905		1.0842
O48-H55	0.9642			C51-C53	1.4012		1.3948
C49-N53	1.1585		1.1519	C54-H55	1.1138		1.0967
C50-N54	1.1546		1.1517	C54-H56	1.1101		1.0882
				C54-C57	1.5376		1.5367
				C57-H58	1.1151		1.0943
				C57-H59	1.1106		1.1041

Table S2: Mulliken atomic charges for DDQ, TCNQ, KT, KT→TCNQ and KT→DDQ complexes

Atoms	KT→DDQ	KT	DDQ	Atoms	KT→TCNQ	KT	TCNQ
O1	-0.376	-0.381		N1	-0.359		-0.295
C2	0.332	0.328		N2	-0.356		-0.295
C3	-0.318	-0.325		N3	-0.358		-0.295
S4	0.33	0.31		N4	-0.359		-0.295
C5	-0.28	-0.287		C5	-0.029		-0.023
C6	-0.056	-0.065		C6	-0.036		-0.023
C7	-0.007	0.018		C7	-0.105		-0.028
C8	-0.049	-0.082		C8	-0.086		-0.028
C9	-0.09	-0.066		C9	-0.085		-0.028
C10	-0.085	-0.094		C10	-0.088		-0.028
C11	-0.083	-0.084		C11	-0.003		0.137
C12	-0.104	-0.11		C12	-0.018		0.137
C13	-0.104	-0.11		C13	0.076		0.111
C14	-0.061	-0.076		C14	0.073		0.111
C15	-0.293	-0.291		C15	0.094		0.111
C16	-0.082	-0.024		C16	0.096		0.111
C17	-0.275	-0.154		H17	0.138		0.155
C18	-0.046	-0.144		H18	0.125		0.155
N19	-0.345	-0.415		H19	0.159		0.155
C20	-0.197	-0.226		H20	0.126		0.155
C21	0.257	-0.027		C21	-0.22	-0.226	
C22	-0.218	-0.343		H22	0.177	0.131	
H23	0.176	0.154		H23	0.196	0.103	
H24	0.136	0.148		H24	0.18	0.129	
H25	0.116	0.113		N25	-0.296	-0.415	
H26	0.124	0.121		C26	0.244	-0.144	
H27	0.125	0.122		H27	0.222	0.142	
H28	0.118	0.116		C28	-0.288	-0.154	
H29	0.209	0.201		H29	0.217	0.154	
H30	0.155	0.152		H30	0.246	0.148	
H31	0.187	0.154		C31	-0.029	-0.024	
H32	0.179	0.148		C32	-0.048	-0.082	
H33	0.182	0.12		C33	-0.011	0.018	
H34	0.201	0.142		C34	-0.06	-0.065	
H35	0.176	0.131		H35	0.139	0.141	
H36	0.16	0.103		C36	-0.275	-0.287	
H37	0.18	0.129		H37	0.177	0.173	
H38	0.196	0.139		S38	0.314	0.31	
H39	0.209	0.17		C39	-0.288	-0.325	

H40	0.21	0.157		C40	0.335	0.328	
C41	-0.08		0.038	O41	-0.374	-0.381	
C42	0.02		0.039	C42	-0.271	-0.291	
C43	0.316		0.447	H43	0.157	0.152	
C44	-0.189		-0.183	C44	-0.087	-0.094	
C45	-0.217		-0.183	C45	-0.076	-0.084	
C46	0.367		0.446	H46	0.118	-0.084	
O47	-0.486		-0.263	C47	-0.125	-0.11	
O48	-0.367		-0.263	H48	0.126	-0.11	
C49	0.119		0.142	C49	-0.116	-0.11	
C50	0.104		0.142	H50	0.128	-0.11	
Cl51	-0.031		-0.264	C51	-0.058	-0.076	
Cl52	-0.022		0.083	H52	0.125	-0.076	
N53	-0.391		0.083	C53	-0.098	-0.066	
N54	-0.324		-0.264	C54	-0.227	-0.343	
H55	0.291			H55	0.192	-0.343	
				H56	0.182	0.17	
				C57	-0.136	-0.027	
				H58	0.204	-0.027	
				H59	0.199	0.139	
				H60	0.196	0.113	

Table S3. Calculation UV-Vis of KT, KT^{+1}, DDQ, HDDQ·, TCNQ, TCNQ^{-1}·, KT^{+1}→TCNQ^{-1}· and KT^{+1}→HDDQ· by TD – DFT/ MPW1PW91 methods with 6-311++ G(d,2p) basis sets.

Molecule	Total Energy, E(TD-HF/TD-KS)	Excited State		E (ev)	λ (nm)	F
KT		78 -> 83<br>80 -> 83<br>81 -> 83<br>82 -> 83	0.27026<br>0.18579<br>0.50139<br>0.35285	3.6335	341.23	0.0070
		80 -> 83<br>81 -> 83<br>82 -> 83	-0.18556<br>-0.3256<br>0.58701	4.0862	303.42	0.2031
		78 -> 83<br>79 -> 83<br>80 -> 83<br>81 -> 83	0.24997<br>0.13566<br>0.539<br>-0.33445	4.2963	288.58	0.0394
(KT-H)$^{+1}$	-1262.76	77 -> 82<br>77 -> 83<br>79 -> 82<br>79 -> 83<br>81 -> 82<br>81 -> 83	0.19761<br>-0.1817<br>0.12855<br>-0.11482<br>0.47586<br>-0.37577	3.6532	339.38	0.0053
		80 -> 82<br>80 -> 83<br>81 -> 82<br>81 -> 83	0.33033<br>-0.33069<br>0.41359<br>0.2668	4.2666	290.59	0.0768
HDDQ^{-1}	-1485.72	57A -> 58A<br>57B -> 58B<br>57A <- 58A<br>57B <- 58B	0.713<br>-0.713<br>0.12684<br>-0.12684	1.6188	765.89	0.0000
		57A -> 58A<br>57B -> 58B	0.70579<br>0.70579	2.9362	422.27	0.2460
		54A -> 58A<br>55A -> 58A<br>57A -> 59A<br>54B -> 58B<br>55B -> 58B<br>57B -> 59B	-0.10786<br>0.35488<br>0.58888<br>0.10786<br>-0.35488<br>-0.58888	3.0021	412.99	0.0000
DDQ	-1485.06	56 -> 57	0.70219	2.7080	457.85	0.0122
		55 -> 57	0.69324	2.8024	442.42	0.0001
		53 -> 57	0.69175	2.9977	413.60	0.0000
TCNQ	-678.68	52 -> 53<br>52 <- 53	0.71677<br>-0.14069	2.9192	424.71	1.2200
		51 -> 53	0.70615	3.2768	378.37	0.0000
		50 -> 53		4.3477	285.17	0.0000

$TCNQ^{-1\cdot}$	-678.68	53A -> 55A<br>52B -> 53B	0.19899<br>0.97549	1.7068	726.40	0.4111
		52A -> 54A<br>51B -> 53B<br>52B -> 54B	0.10966<br>0.97897<br>-0.1179	2.9092	426.18	0.0000
		53A -> 54A	0.98545	3.4459	359.80	0.0048
KT-TCNQ	-1941.54	134A ->135A	0.99990	1.4151	876.16	0.0000
		134A ->139A<br>134A ->140A<br>133B ->134B	-0.16739<br>0.1009<br>0.97376	1.7112	724.52	0.3245
		133B ->135B	0.99916	2.1865	567.05	0.0000
KT-DDQ	-2748.76	138 ->139	0.70644	3.0156	411.14	0.0004
		138 ->140	0.70208	3.1360	395.36	0.1984
		138 ->141	0.70368	3.2743	378.66	0.0032
